# Proteomic analysis of reserve proteins in commercial rice cultivars

**DOI:** 10.1002/fsn3.1375

**Published:** 2020-02-25

**Authors:** Sara Graziano, Nelson Marmiroli, Mariolina Gullì

**Affiliations:** ^1^ Interdepartmental Center SITEIA.PARMA University of Parma Parco Area delle Scienze Parma Italy; ^2^ Department of Chemistry Life Sciences, and Environmental Sustainability University of Parma Parma Italy

**Keywords:** 2D‐GE, allergens, *Oryza sativa*, seed storage proteins

## Abstract

Rice consumption is rising in western countries with the adoption of new nutritional styles, which require the avoidance of gluten. Nevertheless, there are reports of rice allergic reactions. Rice grains contain a low amount of proteins most of which are storage proteins represented by glutelins, prolamins, albumins, and globulins. Some of these proteins are seed allergenic proteins as α‐amylase/trypsin inhibitor, globulins, β‐glyoxylase, and several glutelins. Italy is the major rice producer in Europe, and for this, seed reserve proteins of four Italian rice cultivars were characterized by 2D‐GE analysis. Some differentially abundant proteins were identified and classified as allergenic proteins, prompting a further characterization of the genes encoding some of these proteins. In particular, a deletion in the promoter region of the 19 KDa globulin gene has been identified, which may be responsible for the different abundance of the protein in the Karnak cultivar. This polymorphism can be applied for cultivar identification in commercial samples. Seed proteome was characterized by a variable combination of several proteins, which may determine a different allergenic potential. Proteomic and genomic allowed to identify the protein profile of four commercial cultivars and to develop a molecular marker useful for the analysis of commercial products.

## Graphical abstract

Commercial rice cultivars have a specific profile of allergenic proteins, suggesting variability in their allergenic potential.

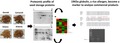

## 1 INTRODUCTION

*Oryza sativa* L. is one of the main food commodity, and nearly half of the world population depend on this culture for daily nutrition. Rice is cultivated mainly in Asia, but in Europe, Italy is the largest producer of rice with an area of 234.133 ha and a production of over 1.5 M ton, representing about 39% and 36% of the harvest area and of the European production, respectively (Faostat, December 2018). Both *japonica* and *indica* varieties are cultivated and, some *japonica* cultivars, such as Arborio, Carnaroli, Vialone nano, are present only in Italy.

Rice consumption is steadily rising in western countries, with the adoption of new nutritional styles and the avoidance of gluten. Rice grains contain a low amount of proteins (7%–10%) most of which are storage proteins represented by glutelins, prolamins (poor in lysine), and a lower amount of albumins and globulins. The aminoacidic composition of seed storage proteins (SSP) contributes to the nutritional quality of rice seeds (Shewry and Halford, 2002). The digestibility and biological value of rice proteins are higher than those of the other major cereals (Amagliani, O’Regan, Kelly, & O’Mahony, 2017).

Rice is generally recognized as a hypoallergenic food, is the first solid food introduced into the diet of infants, and is used in most elimination diets for food allergy diagnostic programs in children and adults. Rice flour represents a common ingredient in the preparation of gluten‐free products like bread and pasta. Rice proteins contributing significantly to the quality and technological functionality of these products (Amagliani et al., 2017). The use of rice proteins as food supplement in sports is also increasing, substituting those commonly used from casein, whey, and soy. Some studies have shown that rice protein concentrates can be used as value‐added ingredients in the production of bread (Jiamyangyuen, Srijesdaruk, and Harper 2005), biscuits (Yadav, Pandey, & Kumar, 2011), and edible films (Adebiyi, Adebiyi, Jin, Ogawa, & Muramoto, 2008) improving their nutritional and functional properties.

Nevertheless, there are some cases in which rice allergic reactions have been reported mainly in Japan and less frequently in Europe and the USA (Goliáš et al., 2013; González‐De‐Olano et al., 2012; Kumar et al., 2007; Villalta, Longo, Mistrello, Amato, & Asero, 2012). Rice allergy is more prominent in adults than in children (Kumar et al., 2007; Birla et al., 2017). The rice seed proteins responsible for allergy are α‐amylase/trypsin inhibitor (14–16 kDa) classified as albumins, α‐globulins, β‐glyoxylase, and several glutelins (Adachi et al., 1993; Birla et al., 2017; Usui et al., 2001).

At variance with other cereals, rice seed proteome is made mainly of glutelins (60% to 80%) that are encoded by 34 genes, while only 5% is represented by prolamins that are encoded by 34 genes (Kawakatsu, Hirose, Yasuda, and Takaiwa 2010). SSP are stored in rice endosperm cells within protein bodies (PB); in particular, glutelins and globulins are deposited in PB‐II storage vacuoles, whereas prolamins accumulate in the endoplasmic reticulum(ER)‐derived protein body I (PB‐I) structures that form within the lumen of the rough ER (Kim, Lee, Yoon, Lim, & Kim, 2013; Saito et al., 2008). Some proteins encoded by single gene have also been identified as the seed allergenic proteins RAG2 and RA5, globulin 19 KDa (Goliáš et al., 2013), or the 56 kDa gluten‐bound starch synthase I (GBSSI) (Krishnan & Chen, 2013).

Rice glutelin originates from the same ancestral gene as 11S globulin, and it is synthesized as a Mr 57000 (57 kDa) precursor protein and transported into the PB‐II via ER and Golgi or directly from ER, where it is processed into mature Mr 37000 (37 kDa) acidic and Mr 20000 (20 kDa) basic subunits (Furuta, Yamagata, Tanaka, Kasai, & Fujii, 1986; Krishnan & Okita, 1986; Yamagata & Tanaka, 1986). Glutelins can be classified into four groups (GluA, GluB, GluC, and GluD) based on their similarity of their amino acid sequence (Kawakatsu, Yamamoto, Hirose, Yano, & Takaiwa, 2008).

Prolamins are classified into three groups (10, 13, and 16 kDa) according to their mobility on SDS‐PAGE gels.

Rice was the first crop to have its genome publicly available (International Rice Genome Sequencing Project & Sasaki, 2005), giving the opportunity of developing functional genomics tools as proteomics, invaluable to assess global changes in protein profiles (Agrawal and Rakwal, (2006); Agrawal & Rakwal, 2011; Hirano et al., 2016).

This study used two‐dimensional gel electrophoresis (2D‐GE), to examine the proteomic profile of mature seeds of four Italian rice cultivars (cvs). The cvs analyzed belong to different rice commercial groups: Carnaroli and Karnak are of the Carnaroli group; Arborio and Volano are of the Arborio group. Our specific goals were to compare the proteomic profile of SSP in these Italian cvs and to verify the expression of those proteins considered as allergens. Differentially abundant spots were identified by matrix‐assisted laser desorption/ionization time‐of‐flight mass spectrometry (MALDI‐TOF‐MS) or by ORBITRAP MS/MS analysis, and eight of them are classified as allergens. The analysis at genomic level revealed the presence of different allelic forms; in particular, in Karnak the gene encoding the 19 kDa globulin carries a deletion in the promoter region. This may explain the more abundant protein expression (twofold) observed in Karnak in comparison with Carnaroli.

## 2 MATERIALS AND METHOD

### 2.1 Plant materials

Seeds of *O. sativa L. spp. japonica*, cvs Arborio, Volano, Carnaroli, and Karnak, were kindly provided by the Rice Research Unit, CREA (Vercelli, Italy). The main characteristics of each cvs are reported in Table S1. Commercial samples of Carnaroli and Karnak were purchased from local market. For both proteomics and genomics analyses, seeds were milled with the refrigerated sample mill Knifetec™ 1095 (Foss) to obtain a fine powder.

#### 2.1.1 Protein extraction

Proteins were extracted from rice flours under denaturing conditions according to Khan et al. (2008) with minor modification. Fifty mg of rice flour was added with 1.4 ml of extraction buffer containing 125 mM tris(hydroxymethyl)aminomethane Tris‐HCl pH 6.8, 8 M Urea, 4% (w/v) sodium dodecyl sulfate SDS, 5% (v/v) β‐mercaptoethanol, and 20% (w/v) glycerol. Samples were vortexed vigorously and incubated at room temperature with overnight agitation. Supernatants were collected after centrifugation at 12,000 g for 15 min at room temperature and then precipitated with 4 volumes of cold acetone. Pellets were dissolved in ¾ volumes of water and ¼ volumes of cold trichloroacetic acid 50% (v/v). Samples were kept in ice bath for 30 min and centrifuged at 12,000 g for 15 min at 4°C. Pellets were rinsed with cold acetone for three times.

### 2.2 2D‐GE analysis

The pH 3–10 IPG strips (Bio‐Rad®) were rehydrated with 85 µl of a buffer containing 30 µg of protein samples, and the rehydration solution composed by 9M urea, 4% (w/v) 3‐[(3‐cholamidopropyl)dimethyl‐ammonio]‐1‐propanesulfonate (CHAPS), 2% (v/v) immobilized pH gradient (IPG) buffer pH 3–10 (Bio‐Rad®), 1% (w/v) bromophenol blue, and 1.2% (v/v) DeStreak Reagent (GE Healthcare). The rehydration step was carried out for 12 hr at 20°C. Isoelectro‐focusing (IEF) was carried out using a Protean® i12™ IEF System (Bio‐Rad®) according to the following program: 250 V for 1 hr, a linear ramp to 4,000 V for 1 hr, and finally 4,000 V to reach 15 kV.

The pH 6–11 IPG strips (GE Healthcare) were rehydrated for 12 hr at 20°C in 135 µl of rehydration solution previously reported with specific buffer pH 6–11 (GE Healthcare). A 100 µg of protein extract was loaded using the cup‐loading method. IEF was carried out using a Protean® i12™ IEF System (Bio‐Rad®) according to the following program: 150 V for 2 hr, 300 V for 2 hr, 600 V for 2 hr, 1,000 V for 1 hr, a linear ramp to 4,000 V for 1 hr, and 4,000 V to reach 15,000 V.

After IEF, the strips were equilibrated for 15 min with agitation in an equilibration solution containing 6 M Urea, 1.5 M Tris‐HCl pH 8.8, 10% (w/v) SDS, 20% (w/v) glycerol, and 2% (w/v) dithiothreitol (DTT) and then in the same equilibration solution containing 2.5% (w/v) iodoacetamide for additional 15 min.

Strips were embedded in 0.5% (w/v) agarose on the top of a 15% acrylamide gel. SDS‐PAGE was performed in a Mini‐PROTEAN® Tetra cell apparatus (Bio‐Rad®) at 150 V, in a running buffer containing 0.125 M Trizma base, 1.25 M Glycine, and 10% (w/v) SDS. Molecular Weight Marker® (Mw 14,000–66,000) (Sigma‐Aldrich) was used. The gels were stained with brilliant blue G‐colloidal solution (Sigma‐Aldrich), then fixed in 7% (v/v) acetic acid and 40% (v/v) methanol, and destained in 25% (v/v) methanol. Three replicates from each sample were analyzed by 2D‐GE.

### 2.3 Image and data analysis

For each sample, a master gel was obtained including all reproducible spots from three replicate gels. In particular, gels were analyzed by ChemiDoc™ MP System (Bio‐Rad®) with Image Lab Software 4.0 (Bio‐Rad®). PDQuest 8.0 2D‐GE Analysis Software (Bio‐Rad®) was used to compare three 2D‐GE raw gels (biological replicates) for each rice cultivar. The analysis included spot detection, background subtraction, gel matching, generation of a master gel, and relative quantification of each spot. The spot intensity in each master map is proportional to the amount of protein in replicate gels and is normalized to the total protein fraction. To quantify differences between the samples, a threshold fold variation window was set at ±2 and Student's *t* test *p*‐value < .05.

### 2.4 Protein identification

For in‐gel digestion, protein spots were removed from the 2D‐GE with a razor blade and destained in a solution of 100 mM 1:1 (v/v) ammonium bicarbonate/acetonitrile (ACN) over night. Proteins were digested in a solution of 10 mM NH_4_HCO_3_ and 10% (v/v) ACN containing trypsin (13 ng/µl) at 37°C overnight. The digested peptides were then suspended in 10 μl of 0.1% trifluoroacetic acid and purified with a ZipTipC18 (Merck Millipore) using the procedure recommended by the manufacturer. Matrix‐assisted laser desorption/ionization time‐of‐flight mass spectrometry (MALDI‐TOF/MS) analysis in linear mode was carried out as previously described (Visioli et al., 2016) using a 4800 Plus MALDI‐TOF/TOF™ (AB SCIEX). Three biological replicates for each sample were performed.

Peptides were also analyzed by ORBITRAP MS/MS using the system LTQ Orbitrap XL (Thermo Fisher Scientific), as previously reported (Graziano et al., 2019). This analysis was performed either as a confirmation of the MALDI/TOF results or as an alternative when MALDI/TOF failed.

### 2.5 Genomic DNA extraction

Genomic DNA was extracted from 300 mg of Carnaroli and Karnak flours using the GK‐Resin method as previously reported (Pafundo, Gullì, & Marmiroli, 2011). DNA yields were determined spectrophotometrically using a VARIAN Cary®50 UV‐VIS device (Agilent Technologies), by measuring the absorbance (A) at 260 nm. Quality of DNA was estimated by agarose gel electrophoresis and by evaluating the ratio A_260_/ A_280_.

### 2.6 PCR conditions and amplicon analysis

Endpoint PCR was carried out in a final volume of 20 μl containing 50 ng of DNA in the presence of 1× Taq buffer (Qiagen), 0.25 μM of each forward and reverse primer, 0.2 mM dNTPs, 2 mM MgCl_2_, and 2.5 U of HotStartTaq DNA polymerase (Qiagen), in a Veriti 96‐well thermal cycler (Applied Biosystems). PCR conditions were as follows: 5‐min initial denaturation at 95°C followed by 35 cycles with a 30 s denaturation at 95°C, 45 s of annealing at 60°C, 1 min and 30 s of elongation at 72°C, and 5 min of final extension at 72°C. Amplification products were analyzed by electrophoresis on 3% (w/v) agarose gel and stained with GelRed Nucleic Acid Gel Stain 1000X (Biotium). The PCR products of the expected size were recovered from agarose gel using a Zymoclean Gel DNA Recovery kit (Zymo Research) or directly from PCR reaction using a CleanSweep™ PCR Purification (Applied Biosystems). The nucleotide sequence of the selected PCR products was determined using gene‐specific primers through an external service (BMR Genomics).

### 2.7 Bioinformatics analysis

#### 2.7.1 Proteomic data

Peptide mass fingerprinting was carried out using the Mascot program (http://www.matrixscience.com). Proteins were identified by searching through the SWISS‐PROT and NCBI nonredundant databases (limited to *Oryza sativa*).

Raw data obtained by ORBITRAP MS/MS were submitted to Proteome Discoverer (Thermo Fisher Scientific) against *Viridiplantae* database (http://www.expasy.org), *Oryza sativa* was chosen for the taxonomic category, and 0.2 Da was used as the mass error tolerance. In both cases, the search parameters were as follows: trypsin as digestion enzyme with a maximum of two missed cleavage; carbamidomethylation of cysteines (delta mass: 57.0215) as static side chain modification; oxidation of methionine (delta mass, 15.9994) and deamination of asparagine, glutamine, and arginine as dynamic side chain modification (delta mass, 0.9840); precursor mass tolerance 10 ppm; and fragment mass tolerance 0.8 Da.

The relative abundance of the protein spots was visualized using Heatmapper, a freely available web server (Babicki et al., 2016).

#### 2.7.2 Genomic data

Sequence information of the gene encoding the 19 KDa globulin was retrieved from Ensembl Plants (http://plants.ensembl.org/Oryzasativa/Transcript) (Fig. S1). Specific primers were designed using the software Primer3 (http://bioinfo.ut.ee/primer3-0.4.0). Primers specificity was verified in silico, using the BLAST program (http://blast.ncbi.nlm.nih.gov/). Primers were purchased from Sigma‐Aldrich. The information about target genes and primers characteristics is reported in Table 1. The sequences of amplicons were analyzed using the BlastN algorithm (http://blast.ncbi.nlm.nih.gov/) and compared using ClustalW (https://www.ebi.ac.uk/Tools/msa/clustalo/).

**Table 1:** Target gene selection and primers design

Protein name (Uniprot Accession number)	Target gene accession number	Primer name	Primer sequence 5’−3’	Amplicon length (bp)
RAG2 (Q01882)	Os07g0214300	RAG2_P	Forward CACCCCATGCTAACAACCCT Reverse TGGATTGATATGCACCACACTCT	596
		RAG2_O	Forward TGAACAGCTAAAACTTTTATGTCCCT Reverse TTTGCAAAATCCGCACTCCG	926
RA5 (Q01881)	Os07g0215500	RA5_P	Forward ATTGATTTCACAGGATTGAGCTC Reverse TGGTCTTGGTGGTGGTACTC	765
		RA5_O	Forward TGTGTCCGTTCTTGAGATCACT Reverse CACACCACATACATGCACAGC	633
19 KDa‐globulin (P29835)	Os05g0499100	19kDa_P	Forward TTTCGGATCGAATTGCCACG Reverse GGCGATCGGATGGGGTTTTA	749
		19kDa_O	Forward TAAAACCCCATCCGATCGCC Reverse TAGCTATGAGGCAAGCCAGC	741
19 KDa‐globulin	MK890106 (Carnaroli) MK890107 (Karnak)	19B	Forward TGAGCCATATATACCGTGGGC Reverse CCTAGGGGTTGCTTGTTGG	123 (Karnak) Null (Carnaroli)
18S rRNA	AH001749	18S	Forward GTGACGGAGAATTAGGGTTCGA Reverse CCGGTATTGTTATTTATTGTCACTACCTC	131

Note

For each primer pair the following information are given: protein name, target gene accession number, primer name and sequence (5′–3′), and amplicon length (bp).

The promoter sequences were analyzed with the PlantPAN 2.0 software (http://plantpan2.itps.ncku.edu.tw) which identifies all the regulatory elements present.

## 3 RESULTS AND DISCUSSION

### 3.1 Proteomic profile and protein identification in rice seeds

**Figure 1:**
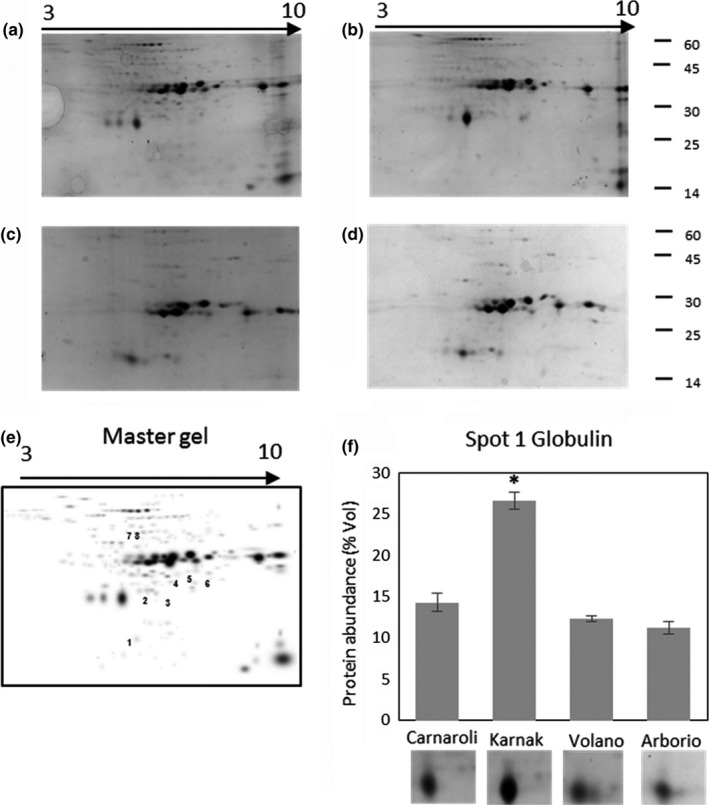
2D‐GE profiles of proteins extracted from rice flours of Carnaroli (a), Karnak (b), Volano (c), Arborio (d), and master gel (e). Proteins were focused on IPG strips pH3‐10, and differentially abundant spots are numbered on the master gel. (f) The relative abundance of spot 1 was evaluated in the four cultivars. *Significant difference (*p* < .05)

**Figure 2:**
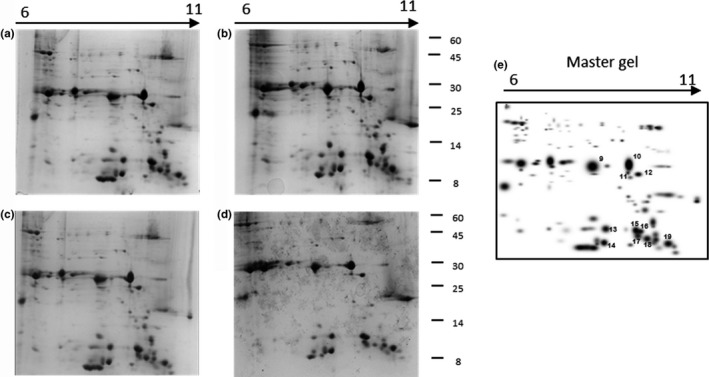
2D‐GE profiles of proteins extracted from rice flours of Carnaroli (a), Karnak (b), Volano (c), Arborio (d), and master gel (e). Proteins were focused on IPG strips pH 6–11, and differentially abundant spots are numbered on the master gel

Proteomic analysis of seed proteins of four Italian rice cvs was carried out by 2D‐GE. In particular, protein extracts were initially separated across a broad‐range IGP strips (pH 3–10) (Figure 1) and then across midrange strips (pH 6–11) (Figure 2) to allow a better separation of basic proteins. In total, 73 spots were identified in pH 3–10 strips and 95 spots in pH 6–11 strips, of which 19 spots resulted differentially abundant (Table 2). The basic range IPG strips associated with cup‐loading method allowed a better separation of basic proteins, ranging from 10 to 60 kDa.

**Table 2:** Identification of highly abundant protein spots by MALDI‐TOF/MS and ORBITRAP MS/MS

Spot	Protein name	Pt Mascot (% Coverage)	Pt Discoverer (% Coverage)	MW (kDa) (theo)	pI (theo)	Uniprot accession
1	19 KDa Globulin	59 (36)		18,9	6,57	P29835
2	Glutelin A3 (acidic/basic)	56 (36)		31,9/21,5	6,1/9,7	Q09151
3	Glutelin A1 (acidic/basic)	54 (35)		31,8/21,7	6,6/9,7	P07728
4	Glutelin A2 (acidic/basic)	78 (37)		31,9/21,7	6,6/9,7	P07730
5	Glutelin B4/B5	36 (20)	343 (42)	32/22,1	7,1/9,5	P14614; Q6ERU3
6	Glutelin		160 (37,63)	54,8	9,02	Q9ZWJ8
7	GBSSI	76 (51)		58,5	6,10	Q0DEV5
8	GBSSI	91 (39)		58,5	6,10	Q0DEV5
9	Glutelin B2		263 (41,4)	31,2/22,1	7,9/9,5	Q02897
10	Glutelin B1		334 (44,8)	31,7/22,1	8,44/9,69	P14323
11	Glutelin	76 (51)		56,5	9,26	T1T6C4
12	Glutelin C	91 (39)	97.82 (25.6)	53,5	9,39	M1G949
13	Trypsin/alpha‐amylase inhibitor RAG2			15,2	7,69	Q01882
14	17kDa alpha‐amylase/trypsin inhibitor 2	36 (63)	189 (76)	16,4	7,48	Q7X8H9
15	Seed allergenic protein RA5		23 (61)	14,7	8,13	Q01881
16	Hypothetical protein similar to Seed allergenic protein RA17 (RA16)		62 (61)	16,9	8,36	Q8H4L8
17	Seed allergenic protein RAG2 precursor	17 (40)	13,6 (25)	17,3	8,59	Q40653
18	Prolamin PPROL 17D	78 (68)	20 (27)	15,5	8,20	P20698
19	Seed allergenic protein RA5		62 (64)	14,7	8,13	Q01881

Note

For each spot, the following information are reported: the % of total protein coverage, the MASCOT score (for MALDI‐TOF/MS), the Proteome Discoverer score (for ORBITRAP MS/MS), MW and pI, and the UniProt accession number of the matching protein.

Among the differentially expressed spots, the most abundant were identified as different members of the glutelin family (spots 2–6, 9–12, Figures 1e and 2e) and as globulin (spot 1, Figure 1f).

**Figure 3:**
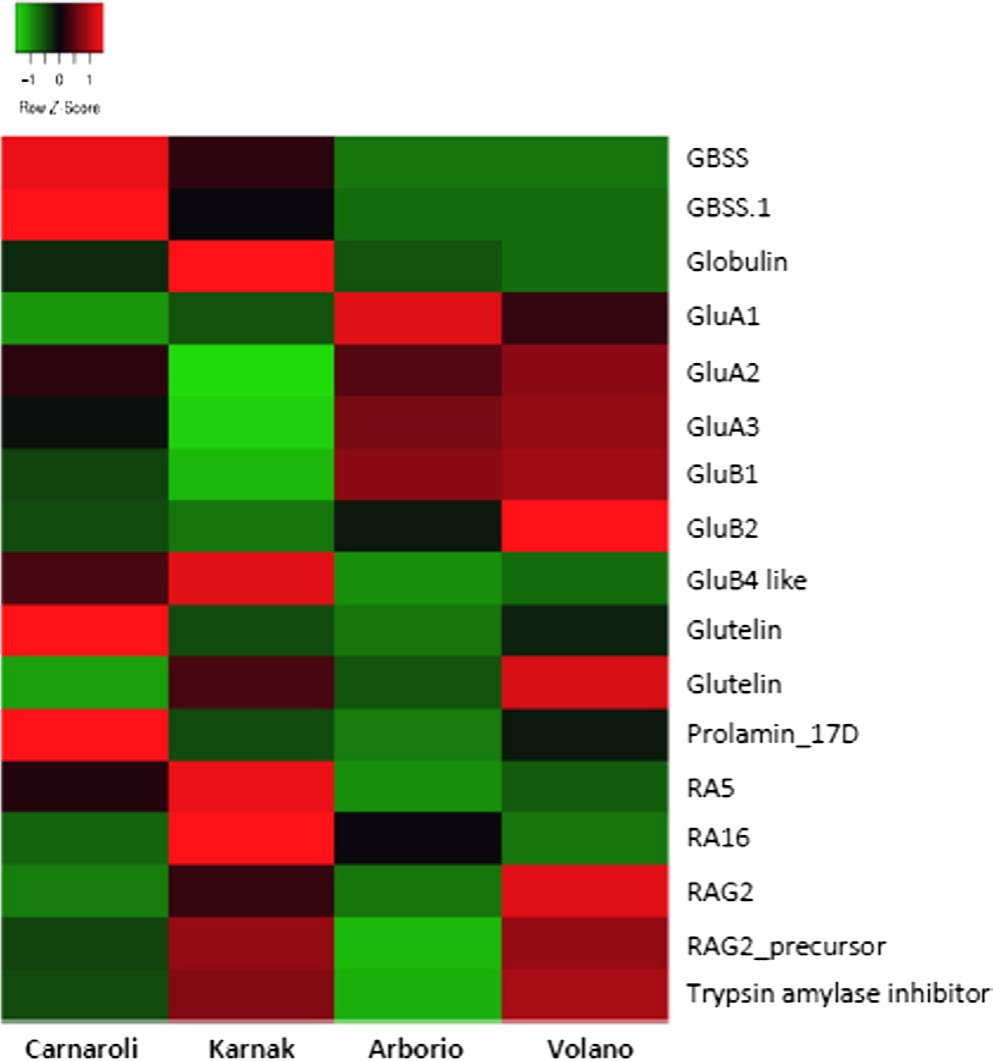
Heat map representing the relative abundance of the protein spots identified by 2D‐GE analysis of rice seed proteins purified from four rice cultivars

The relative amount of each spot was compared in the four samples, and it was observed that five glutelins (spots 2, 3, 4, 9, and 10) were more abundant in Arborio and Volano respect to Carnaroli and Karnak (Figure 3). In particular, GluA2 (spot 4) had the lowest amount in Karnak, and GluB2 (spot 9) was significantly more abundant in Volano.

The isoforms of GBSSI (spots 7–8 in Figure 1e) were more abundant in Carnaroli and Karnak as compared to Volano and Arborio, in which they were almost undetectable on the gel. This enzyme, which is encoded by the *Waxy* gene, regulates the elongation of amylose chain in developing seeds, and the level of grain amylose is directly associated with the amount of GBSSI in the endosperm (Mikami et al., 2008), and differences in the abundance of GBSSI are directly related to rice cooking quality. Arborio and Volano have an apparent amylose content (AAC) of about 17.4% as compared to Carnaroli and Karnak, which have 22.1% and 20.9%, respectively (data from Ente Nazionale Risi). Therefore, according to the suggested commercial classification, they are low (<20%) or medium (21%–25%) AAC (Biselli et al., 2014).

The differences observed are strictly related to the genetic characteristics of Wx sequence; in particular, Karnak is characterized by the allelic pattern G/C (intron 1/exon 6 SNPs), while Arborio has the T/A pattern (Biselli et al., 2014).

Furthermore, eight spots of low molecular weight were identified as allergenic proteins (Table 2) and they resulted differentially abundant among the cvs analyzed (Figure 3). In particular, spot 1, identified as the 19 kDa globulin, was significantly more abundant (twofold) in Karnak respect to the other samples (Figure 1f). RA5 (spots 15, 19) and RA16 (spot 16) were more abundant (threefold and twofold respectively) in Karnak. RAG2 (spot 13), RAG2_precursor (spot 17), and trypsin amylase inhibitor (spot 14) were twofold more abundant in Karnak and Volano as compared to Carnaroli and Arborio. Prolamin_17D (spot 18) was twofold more abundant in Carnaroli.

The globulins of rice have not enjoyed the same level of attention as the prolamins and glutelins. To date, four globulins of molecular weight 16 kDa, 25 kDa (Komatsu & Hirano, 1992; Krishnan & White, 1992), 26 kDa (Nakase et al., 1996), and 19 kDa (Krishnan & Pueppke, 1993; Shorrosh et al., 1992) have been isolated from rice grain endosperm. Each of these proteins is expressed as a precursor, which is processed to generate the mature protein. DNA sequence analysis of the 26 kDa globulin suggested the protein was very similar to wheat HMW glutenin subunit and barley D hordein (Nakase et al., 1996), while the 19 kDa globulin is most likely encoded by a single‐copy gene (Shorrosh et al., 1992) which is similar to the globulins of wheat, rye, and triticale (Krishnan & Pueppke, 1993). It has also been demonstrated that the 19 kDa globulin is mainly localized in the inner part of the rice grain; therefore, it is present also in the polished rice and can be responsible for the allergic reaction (Satoh, Tsuge, Tokuda, & Teshima, 2019).

The study of the promoter region and the ORF of this gene may be useful in order to identify functional markers for breeding/selection of rice Italian cvs with low allergenic potential.

Rice is commonly regarded as a hypoallergenic cereal; however, it has attracted increasingly public attentions after the first allergic reaction reported in 1979 (Shibasaki, Suzuki, Nemoto, & Kuroume, 1979). Hereafter, a number of clinical cases on rice allergy contracted either by contacting with raw rice, inhaling of rice powders or vapors, or by ingesting of rice have been reported (Zhu et al., 2015).

The allergenic activity of these molecules is not well understood,(Goliáš et al., 2013; Krishnan & Chen, 2013) but several candidates, including the 22 allergens present in databases (IUIS Allergen Nomenclature Sub‐Committee: Allergen Nomenclature; http://www.allergen.org/), were predicted by a genome‐wide search through the rice genome (Satoh et al., 2019).

Among the reported rice allergens, expansin (35 kDa, Ory s 1) and profilin A (14 kDa, Ory s 12), a group of 14–16 kDa proteins contains 11 isoallergens belonging to α‐amylase/trypsin inhibitor family. The 26 and 33 kDa seed proteins were characterized as globulin and glyoxalase I, respectively (Krishnan & Chen, 2013). Goliáš et al. (2013) identified six thermostable putative rice allergens: glutelin C precursor, GBSSI protein, disulfide isomerase‐like 1‐1 protein, hypothetical protein OsI 13,867, putative acid phosphatase precursor 1, and a protein encoded by the locus Os02g0453600.

The α‐amylase/trypsin inhibitor family includes several members like RAG2, and their proteomic profile varies among rice cultivars; their relative abundance was either higher or lower than the reference Nipponbare (Teshima, Nakamura, Satoh, & Nakamura, 2010).

### 3.2 Amplification of genes coding seed storage protein

Among the seed allergenic proteins identified in this study as differentially abundant, RAG2, RA5, and the 19 kDa globulin are encoded by single gene copy. Considering the available genomic sequences, we utilized the primer pairs shown in Table 1 to isolate the corresponding genomic sequences from the four cvs. In particular, for each gene we amplified a portion of the promoter region and the complete open reading frame (ORF). Amplicons with the expected size were sequences from the different cultivars and compared by multiple alignment, including also the *Oryza* reference sequence present in the database.

**Figure 4:**
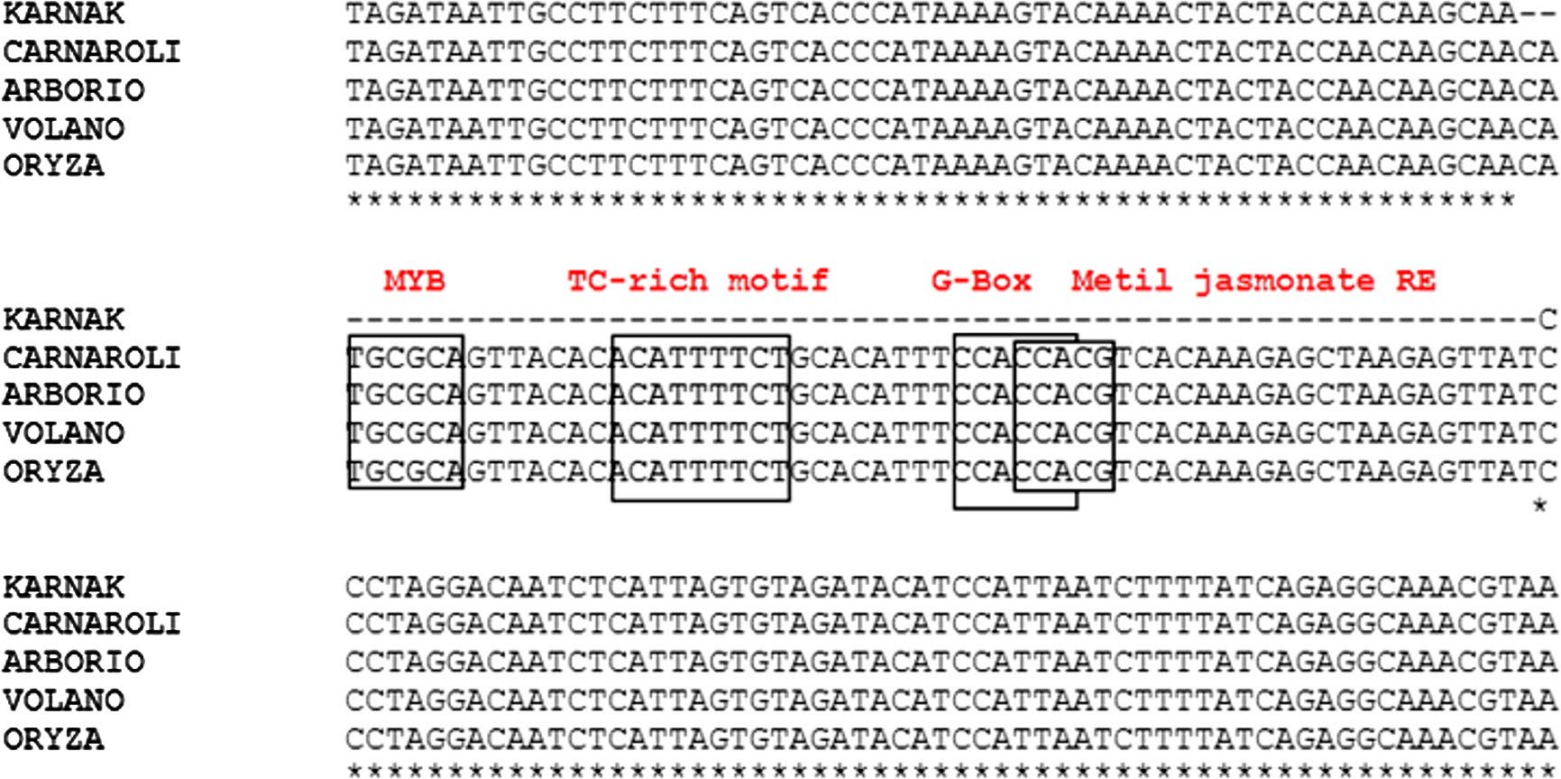
Multiple alignment obtained with CLUSTALW of a region of the promoter of 19 kDa globulin (−533/‐354) obtained from the different rice cvs analyzed using primer pair 19kDaP and compared to the reference sequence of Nipponbare (ORYZA). The boxes indicate the regulatory elements present within the region of 61 bp deleted in Karnak, and the asterisks indicate the conserved bases

Multiple alignment of RAG2 and RA5 revealed an overall conservation especially in the ORFs (data not shown). Multiple alignment of 19 kDa globulin showed a 100% identity in the ORF regions in all cultivars, but some differences in the promoter region. In particular, the promoter region amplified from Karnak has a deletion of 60 bp respect to all the other cvs and the reference sequence of Nipponbare (Figure 4). Within this region, there are several regulatory elements such as Myb binding site, bZIP, Helix‐loop‐helix DNA‐binding domain (bHLH), and TALE which have been predicted on both minus and plus strand (Weirauch et al., 2014). The absence of these sequences could modulate the expression of the 19 KDa globulin gene determining a twofold difference in the abundance of the protein in Karnak respect to Carnaroli, as observed in the 2D‐GE analysis (Figure 1).

**Figure 5:**
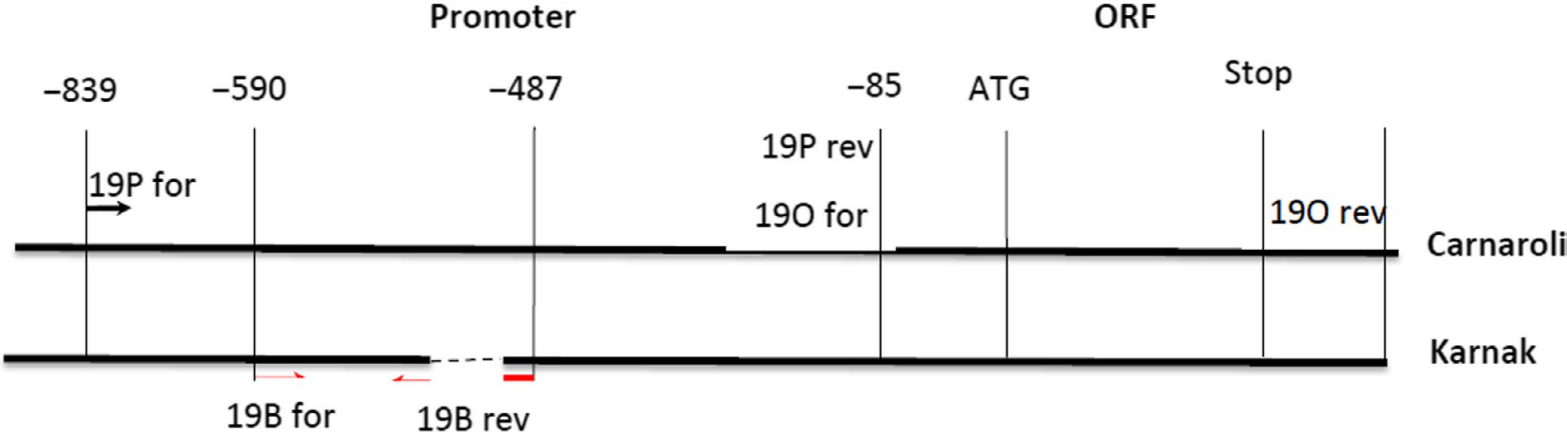
Scheme of the 19KDa globulin gene. The Karnak sequence contains a deletion (interrupted line). The primer pairs used are shown as a pair of arrows of the same shape. Primer pairs 19P and 19O (double and dotted arrows) can amplify both Carnaroli and Karnak DNA, and primer pair 19B (black arrows) can amplify only Karnak DNA. Primers sequences are reported in Table 1

The promoter sequences of the 19 KDa globulin gene were studied in different cvs using different pairs of primers (Figure 5, Table 1). The primer pair 19B_F and 19B_R should allow to discriminate DNA purified from Karnak or Carnaroli, since an amplification product of 123bp was expected only from Karnak DNA. The DNA fragment was sequenced to confirm its identity.

The genomic region on chromosome 5 containing the gene for the 19 kDa globulin harbors a QTL (AQDZ002‐LOI, https://archive.gramene.org) associated with the locus *prl5* for improved lodging resistance.(Kashiwagi & Ishimaru, 2004; Kashiwagi, Madoka, Hirotsu, & Ishimaru, 2006) The cv Karnak is characterized by a higher lodging resistance respect to Carnaroli, and the selection for this improved traits may have determined the coselection for a new quality‐related allele for the 19 kDa globulin with higher expression.

### 3.3 PCR endpoint on commercial products

Samples of rice purchased at local market and identified as "Carnaroli rice" and "Karnak rice" where utilized for the extraction of gDNA. For each sample, three technical replicas were performed and the extracted DNA was then quantified. The amount of gDNA (ng) was 1508.19 ng and 1484.72 ng, for Carnaroli and Karnak, respectively.

Endpoint PCR was performed using the primer pair 19B (Figure S2a) and 18S (Figure S2b) on both commercial samples and on Carnaroli and Karnak control samples. All samples were amplifiable with 18S primers as expected (Figure S2b). Using 19B primers, gDNAs extracted from the Karnak flour and the commercial "Karnak rice" were both amplified resulting in amplicons of the same size (123 bp); differently, the gDNA extracted from the Carnaroli flour did not amplify, while gDNA extracted from the commercial "Carnaroli rice" was amplified showing an amplicon of 123 bp (Figure S2a). This last result may be explained considering that the commercial “Carnaroli rice” could be a mixture of Karnak and Carnaroli grains. Effectively, it is quite difficult to obtain commercial rice with the name Karnak, since in most cases it is labeled as “Carnaroli rice,” which is the common product group, according to Italian regulation.

In conclusion, some proteins showing a significant increase/decrease were identified by mass spectrometry, the most abundant where members of the glutelin family and the globulin. Each cultivar has shown a specific profile of these allergenic proteins, suggesting a variability in their allergenic potential. The genomic analysis of 19 kDa‐globulin, one of the most abundant protein, revealed the presence of a new polymorphism in the promoter region of the Karnak allele, which could explain the different proteomic profile. Since rice is a staple food and an important source of proteins worldwide, a combined proteomic and genomic approach could be justified for a qualitative screening of different cultivars and for developing functional markers for the breeding/selection of rice varieties with lower allergenic potential.

## CONFLICT OF INTEREST

The authors do not have any conflicting interests.

## ETHICAL APPROVAL

This study does not involve any human or animal testing.
