# Supplementary material for: Proteomic analysis of reserve proteins in commercial rice cultivars
Source: Food Sci Nutr. 2020 Feb 25;8(4):1788–97. doi: 10.1002/fsn3.1375 (PMC7174207; doi:10.1002/fsn3.1375)
Supplement: Supplementary file 1 [file FSN3-8-1788-s001.pdf]

Figure S1 Genomic sequence of 19 kDa globulin utilized for primer design. The region of 61 bp deleted in Karnak is bolded.

-985 GCCTGGAGGGAGGAGAGGGGAGAGATGGTGAGAGA
-950 GGAGGAAGAAGAGGAGGGGTGACAATGATATGTGGGGCCATGTGG
-905 GCCCCACCATTTTTTAATTCATTCTTTTGTTGAAACTGACATGTG
-860 GGTCCCATGAGATTTATTATTTTTCGGATCGAATTGCCACGTAAG
-815 CGCTACGTCAATGCTACGTCAGATGAAGACCGAGTCAAATTAGCC
-770 ACGTAAGCGCCACGTCAGCCAAAACCACCATCCAAACCGCCGAGG
-725 GACCTCATCTGCACTGGTTTTGATAGTTGAGGGACCCGTTGTATC
-675 TGGTTTTTCGATTGAAGGACGAAAATCAAATTTGTTGACAAGTTA
-630 AGGGACCTTAAATGAACTTATTCCATTTCAAAATATTCTGTGAGC
-585 CATATATACCGTGGGCTTCCAATCCTCCTCAAATTAAAGGGCCTT
-540 TTTAAAATAGATAATTGCCTTCTTTCAGTCACCCATAAAAGTACA
-495 AAACTACTACCAACAAGCAAC**ATGCGCAGTTACACACATTTTCTG**
-450 ***C*****ACATTTCCACCACGTCACAAAGAGCTAAGAGTTATC**CCTAGGAC
-405 AATCTCATTAGTGTAGATACATCCATTAATCTTTTATCAGAGGCA
-360 AACGTAAAGCCGCTCTTTATGACAAAAATAGGTGACACAAAAGTG
-315 TTATCTGCCACATACATAACTTCAGAAATTACCCAACACCAAGAG
-270 AAAAATAAAAAAAAATCTTTTTGCAAGCTCCAAATCTTGGAAACC
-225 TTTTTCACTCTTTGCAGCATTGTACTCTTGCTCTTTTTCCAACCG
-180 ATCCATGTCACCCTCAAGCTTCTACTTGATCTACACGAAGCTCAC
-135 CGTGCACACAACCATGGCCACAAAAACCCTATAAAACCCCATCCG
- 90 ATCGCCATCATCTCATCATCAGTTCATCACCAACAAACAAAAGAG
- 45 GAAAAAAAACATATACACTTCTAGTGATTGTCTGATTGATCATCA

1 ATGGCTAGCAAGGTCGTCTTCTTCGCGGCGGCGCTCATGGCGGCC
M A S K V V F F A A A L M A A

46 ATGGTGGCCATCTCCGGCGCGCAGCTGAGCGAGTCGGAGATGAGG
M V A I S G A Q L S E S E M R

91 TTCAGGGACAGGCAGTGCCAGCGGGAGGTGCAGGACAGCCCGCTG
F R D R Q C Q R E V Q D S P L

136 GACGCGTGCCGGCAGGTGCTCGACCGGCAGCTCACCGGCCGGGAG
D A C R Q V L D R Q L T G R E

181 AGGTTCCAGCCGATGTTCCGCCGCCCGGGCGCGCTCGGCCTGCGG
R F Q P M F R R P G A L G L R

226 ATGCAGTGCTGCCAGCAGCTGCAGGACGTGAGCCGCGAGTGCCGC
M Q C C Q Q L Q D V S R E C R

271 TGCGCCGCCATCCGCCGGATGGTGAGGAGCTACGAGGAGAGCATG
C A A I R R M V R S Y E E S M

316 CCGATGCCCCTGGAGCAAGGCTGGTCGTCGTCGTCGTCGGAGTAC
P M P L E Q G W S S S S S E Y

361 TACGGCGGCGAGGGGTCGTCGTCGGAGCAGGGGTACTACGGCGAG
Y G G E G S S S E Q G Y Y G E

406 GGGTCGTCGGAGGAGGGCTACTACGGCGAGCAGCAGCAGCAGCCG
G S S E E G Y Y G E Q Q Q Q P

451 GGGATGACCCGCGTGAGGCTGACCAGGGCGAGGCAGTACGCGGCG
G M T R V R L T R A R Q Y A A

496 CAGCTGCCGTCGATGTGCCGGGTTGAGCCCCAGCAGTGCAGCATC
Q L P S M C R V E P Q Q C S I

541 TTCGCCGCCGGCCAGTACTAG
F A A G Q Y *
