# Supplementary material for: Proteomic analysis of reserve proteins in commercial rice cultivars
Source: Food Sci Nutr. 2020 Feb 25;8(4):1788–97. doi: 10.1002/fsn3.1375 (PMC7174207; doi:10.1002/fsn3.1375)

Figure S2

gDNA extracted from flours obtained from commercial products (1,2) or certified seeds (3, 4) of Karnak and Carnaroli respectively, was amplified using (A) 19B primer pairs or (B) 18S primer pair. (M) 100 bp ladder, (5) no template control. Amplicons were separated through a 3% (w/v) agarose gel electrophoresis.

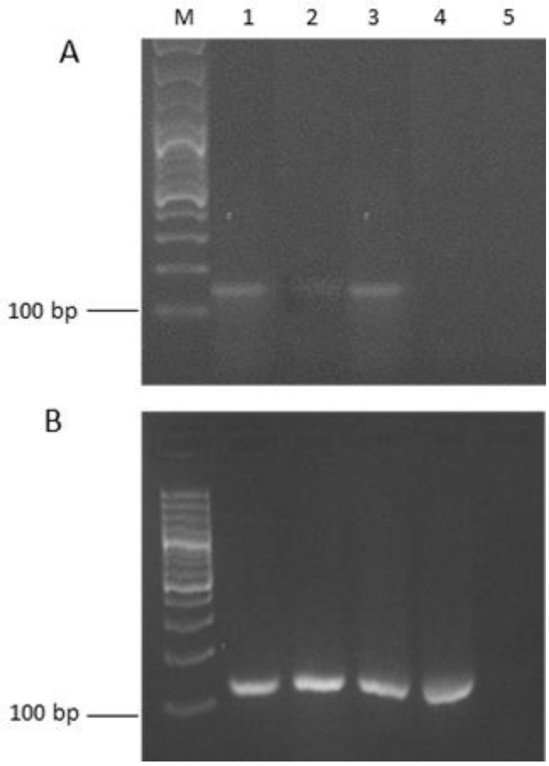

Supplement: Supplementary file 2 [file FSN3-8-1788-s002.pdf]
