# Supplementary material for: Proteomic analysis of reserve proteins in commercial rice cultivars
Source: Food Sci Nutr. 2020 Feb 25;8(4):1788–97. doi: 10.1002/fsn3.1375 (PMC7174207; doi:10.1002/fsn3.1375)
Supplement: Supplementary file 3 [file FSN3-8-1788-s003.pdf]

Table S1. Main agronomic traits and grain quality of rice cultivars utilized.

Rice cultivar	Characteristics	Plant characteristics	Grain characteristics	Tolerance to stress
Arborio	Pedigree Vialone X Lady Wright<br>Year of registration: 1967	Seed sowing: 180-200 kg/ha<br>Sowing period: until 10-15/05<br>Production capacity: medium<br>Pericarp color: white	Length / width ratio: 2,1<br>EU Classification: Long<br>Endosperm: non-glutinous type<br>Amylose: low (17,3 % ss)<br>Consistency: 0,69 kg / cm2<br>Stickiness: 3.03 g x cm	Tolerance to cold in the germination phase: high<br>Tolerance to cold in bloom: discrete
Volano	Pedigree St. 401 X Rizzotto<br>Year of registration: 1972	Seed sowing: 200-220 kg/ha<br>Sowing period: until 10/05<br>Production capacity: medium-high<br>Pericarp color: white	Length / width ratio: 2,1<br>EU Classification: Long<br>Endosperm: non-glutinous type<br>Amylose: low (17, 4 % ss)<br>Consistency: 0.70 kg / cm2<br>Stickiness: 3.23 g x cm	Tolerance to cold in the germination phase: good
Carnaroli	Pedigree Vialone X Lencino<br>Year of registration: 1983	Seed sowing:<br>180-200 kg / ha<br>Sowing period: until 10/05<br>Production capacity: medium-low<br>Pericarp color: white	Length / width ratio: 2.2<br>EU Classification: Long<br>Endosperm: non-glutinous type<br>Amylose: intermediate (22, 1% ss)<br>Consistency: 0.91 kg / cm2<br>Stickiness: 1 g x cm	Tolerance to cold in the germination phase: discrete
Karnak	Pedigree Carnaroli<br>Year of registration: 2002	Seed sowing: 160-180 kg/ha<br>Sowing period: until 10/05<br>Production capacity: medium-high<br>Pericarp color: white	Length / width ratio: 2,1<br>EU Classification: Long<br>Endosperm: non-glutinous type<br>Amylose: intermediate (22,7 % ss)<br>Consistency: 0.96 kg / cm2<br>Stickiness: 1 g x cm	Tolerance to cold in the germination phase: good
